# Homocysteine exchange across skeletal muscle in patients with chronic kidney disease

**DOI:** 10.14814/phy2.15573

**Published:** 2023-03-22

**Authors:** Giacomo Garibotto, Daniela Picciotto, Daniela Verzola, Alessando Valli, Antonella Sofia, Francesca Costigliolo, Michela Saio, Francesca Viazzi, Pasquale Esposito

**Affiliations:** ^1^ Department of Internal Medicine University of Genova Genova Italy; ^2^ Division of Nephrology, Dialysis and Transplantation, IRCCS Ospedale Policlinico San Martino Genoa Italy

**Keywords:** CKD, folate, homocysteine, methionine, muscle

## Abstract

Sites and mechanisms regulating the supply of homocysteine (Hcy) to the circulation are unexplored in humans. We studied the exchange of Hcy across the forearm in CKD patients (*n* = 17, eGFR 20 ± 2 ml/min), in hemodialysis (HD)‐treated patients (*n* = 14) and controls (*n* = 9). Arterial Hcy was ~ 2.5 folds increased in CKD and HD patients (*p* < 0.05–0.03 vs. controls). Both in controls and in patients Hcy levels in the deep forearm vein were consistently greater (+~7%, *p* < 0.05–0.01) than the corresponding arterial levels, indicating the occurrence of Hcy release from muscle. The release of Hcy from the forearm was similar among groups. In all groups arterial Hcy varied with its release from muscle (*p* < 0.03–0.02), suggesting that muscle plays an important role on plasma Hcy levels. Forearm Hcy release was inversely related to folate plasma level in all study groups but neither to vitamin B12 and IL‐6 levels nor to muscle protein net balance. These data indicate that the release of Hcy from peripheral tissue metabolism plays a major role in influencing its Hcy plasma levels in humans and patients with CKD, and that folate is a major determinant of Hcy release.

## Graphical abstract

Sites and mechanisms underlying the supply of Homocysteine (Hcy) to the circulation are unexplored in humans. Methionine synthase is expressed, although at intermediate level, in skeletal muscle, which, however, represents a large part (about 40%) of body weight. At variance with liver, pancreas and kidney, that express the entire methionine cycle, the transsulfuration pathway is virtually absent in muscle; in addition, even if MTHFR is present in muscle, the methionine remethylation pathway is limited by lack of BHMT. We studied the exchange of Hcy across the forearm (which is mainly composed of muscle) in CKD patients, in hemodialysis (HD)‐treated patients and controls. Arterial Hcy was 2.5 folds increased in CKD and HD patients. Both in controls and in patients Hcy levels in the deep forearm vein were consistently greater (+7%, *p* < 0.05–0.01) than the corresponding arterial level, indicating the occurrence of Hcy release from muscle. The release of Hcy from the forearm was similar among groups (−1.8 ± 0.5, −2.1 ± 0.8 and −1.7 ± 0.5 nmol/min). In all groups arterial Hcy varied with its release from muscle, suggesting that muscle plays an important role on plasma Hcy levels. Forearm Hcy release was inversely related to folate level in all study groups. These data indicate that the release of Hcy from peripheral tissue metabolism plays a major role in influencing its plasma levels in humans and patients with CKD, and that folate is a major determinant of Hcy release.

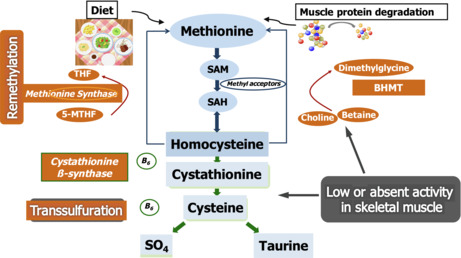

## 1 INTRODUCTION

**FIGURE 1:**
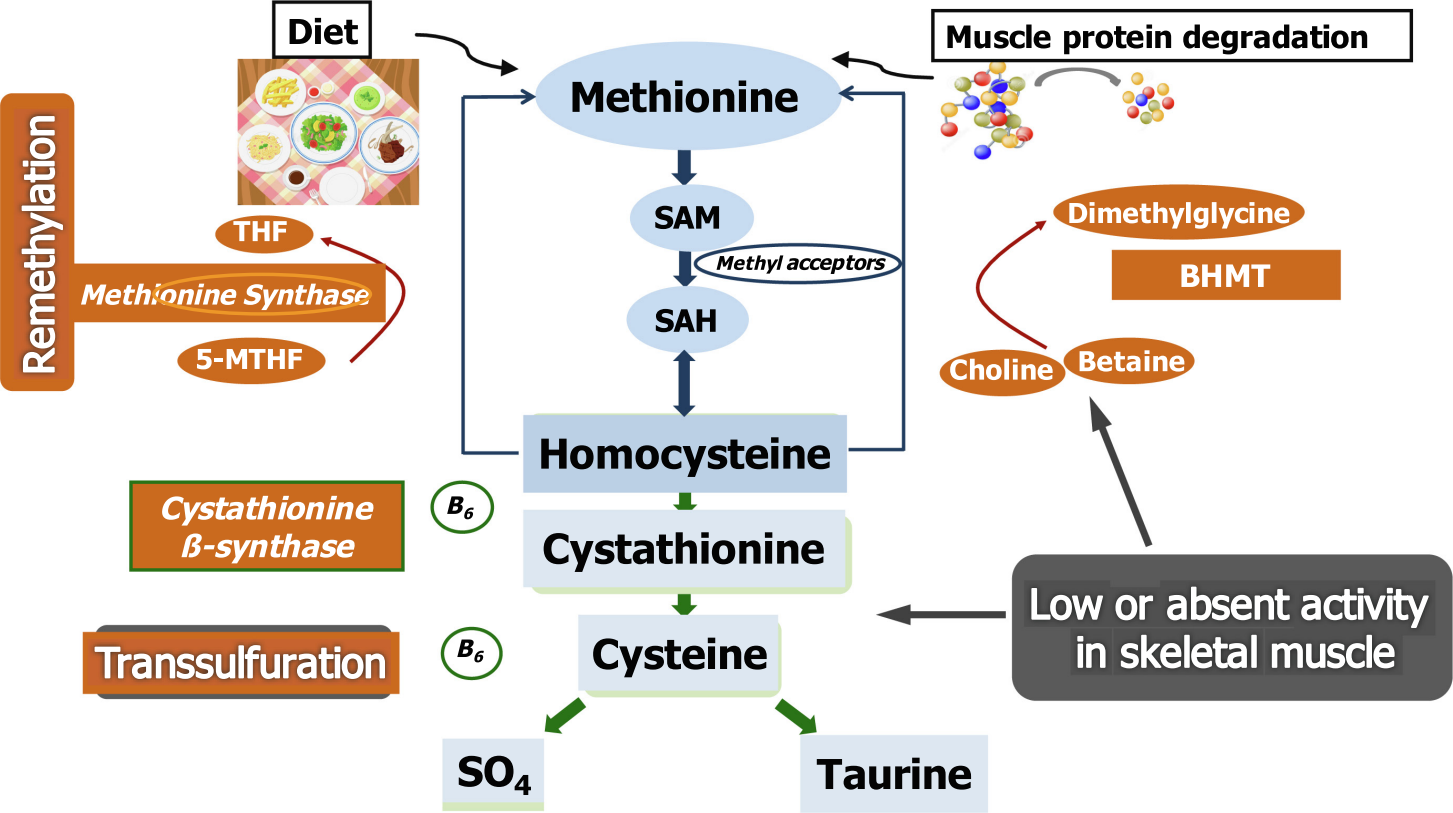
Pathways of methionine metabolism in skeletal muscle. Methionine synthase (MS) is expressed, although at intermediate level, in skeletal muscle. MS and betaine‐homocysteine methyltransferase (BHMT), a zinc metalloenzyme that catalyzes the transfer of methyl groups from betaine to homocysteine (Hcy) to produce dimethylglycine and methionine, are the major enzymes involved in the remethylation pathway. At variance with liver, pancreas and kidney, the transsulfuration pathway is virtually absent in muscle; in addition, the remethylation pathway is limited by lack of BHMT expression.

An increase in plasma homocysteine (Hcy), a putatively atherothrombotic sulfur amino acid, is very common in patients with CKD and occurs almost in 85% of patients with End‐Stage Renal Disease (ESRD) undergoing maintenance hemodialysis (HD; Bostom & Culleton, 1999). Hcy, which is found mainly intracellularly, supports a number of physiological processes, including purine and thymidine biosynthesis, amino acid homeostasis, epigenetic maintenance, and redox defense (Ducker & Rabinowitz, 2017). Tissue Hcy levels reflect a balance between its synthesis from methionine via *S*‐adenosyl‐l‐methionine (SAM)‐dependent methylation reactions, its remethylation to methionine and its catabolism through the transmethylation and transsulfuration pathways (Figure 1). Methionine synthase (MS) and betaine‐homocysteine methyltransferase (BHMT), a zinc metalloenzyme that catalyzes the transfer of methyl groups from betaine to Hcy to produce dimethylglycine and methionine, are the major enzymes involved in the remethylation pathway.

Early studies have shown that patients with CKD have markedly reduced clearance of Hcy from plasma (Guttmorsen et al., 1997). Steady‐state isotopic studies have shown that the flows through the transsulfuration pathway and the remethylation pathway are impaired in patients with CKD (Stam et al., 2003; van Guldener et al., 1999). However, these dynamic whole‐body studies do not reveal the tissue specificity or function of the underlying reactions, and sites and mechanisms which regulate circulating Hcy both in the normal condition and disease are still not completely understood. On a theoretical ground, an increase in plasma Hcy may occur following an increase in its production rate (i.e., transmethylation), a decreased rate of its removal (by transsulfuration or remethylation), or decreased elimination from body fluids.

1‐C pathway enzymes are not uniformly distributed in all tissues. Tissues where transsulfuration or remethylation are restricted may export Hcy for further metabolism by other tissues expressing the entire 1‐C cellular machinery. Early studies have focused 1‐C metabolism in liver, pancreas and kidney (Finkelstein et al., 1971; Mato et al., 2002). In vivo studies in rodents have shown that even if the fraction of intracellular methionine derived from the methylation of Hcy is highest in liver, most Hcy is retained in liver cells (Fiona et al., 2009). In contrast, the pancreas exports to plasma both methionine and Hcy, and this matches matching the contribution from liver (Fiona et al., 2009). In recent years the importance of methionine cycle in skeletal muscle, in particular on inducing differential DNA methylation, has been reappraised both in physiology (Terruzzi et al., 2011) and disease (Van Dyck et al., 2022). Methionine synthase is expressed, although at intermediate level, in skeletal muscle, which, however, represents a large part (about 40%) of body weight. At variance with liver, pancreas and kidney, that express the entire methionine cycle, the transsulfuration pathway is virtually absent in muscle (Figure 1) (Chen et al., 1997; Veeranki & Tyagi, 2015). In addition, the remethylation pathway is mainly dependent on methylenetetrahydrofolate reductase (MTHFR) because of limited expression of both BHMT (Sunden et al., 1997) and the more recently discovered BHMT2 (Chadwick et al., 2000).

In the postabsorptive state and during fasting, skeletal muscle cells meet with a sizable amount of methionine deriving from net protein degradation (Lundholm et al., 1987). Previously, we observed that human leg tissues, which are mainly composed of skeletal muscle, release Hcy in the circulation, while splanchnic organs (which mainly represent liver and gut exchanges) show, as a trend, a positive Hcy balance (Garibotto et al., 2003), a finding which suggests a compartmentalization of 1‐C metabolic reactions to maintain plasma Hcy levels (Garibotto et al., 2003). However, the tissue sources of plasma Hcy in humans have not been identified; in addition, if hyperhomocysteinemia in CKD may in part be due to increased entry into the blood compartment has not been studied yet.

The renewed interest in Hcy as a player in the methylation processes in aging and cachexia (Bauchart‐Thevret et al., 2009; Ducker & Rabinowitz, 2017; Shahal et al., 2022; Terruzzi et al., 2011; Verbruggen et al., 2009) prompted us to review the results of studies on muscle amino acid kinetics performed from our laboratory (Garibotto et al., 2006, 2015). While the results on protein metabolism have been previously published (Garibotto et al., 2006, 2015), data on Hcy exchange are still unpublished. The aim of this study was to evaluate the role of skeletal muscle on the Hcy export to the circulation in CKD patients. In this work we calculated the exchange of Hcy across the forearm, which is mainly composed of muscle, in renal patients and control subjects in from forearm perfusion studies in which Hcy measurements together with forearm blood flow, net protein balance, folate and vitamin B12 data were available (Garibotto et al., 2006, 2015). As a second step, we studied the individual role of muscle net protein balance (as a source of intracellular methionine), interleukin‐6 (IL‐6) (as a marker of inflammation), folate and vitamin B 12 status, on Hcy muscle handling. Taken together our results indicate that skeletal muscle plays a role greater than previously supposed in Hcy production and export to other tissues in fasting conditions, and that Hcy release from muscle is strictly dependent on folate availability.

## 2 MATERIAL AND METHODS

Arterial and forearm deep vein plasma Hcy measurements were available in 17 patients with CKD 4–5 (age 53 ± 6 years, eGFR 20 ± 2 ml/min, 14 M/3F, BMI 25 ± 2), 14 patients with CKD5d on maintenance thrice‐weekly hemodialysis (HD) schedule (age 64 ± 4 years, 11 M/3F, BMI 24 ± 1, Kt/V 1.3 ± 0.10) and 9 control subjects (age 45 ± 4 years, BMI 23 ± 4, 6 M/3F; Garibotto et al., 2006, 2015). Six CKD patients and 7 HD patients displayed clinical signs of cardiovascular disease and increased CRP levels (>5 mg/L). Average protein intake was 0.9 ± 0.1 and 1.1 ± 0.1 g/kg, respectively, in CKD and HD patients; calorie intake was 30–32 and 28–31 kcal/kg, respectively, in each group, as estimated by nutritional interviews. BMI, fat free and fat mass (anthropometric measures) were not different in patients and controls (Garibotto et al., 2006, 2015). Both CKD and HD patients received phosphate binders, sodium bicarbonate, vitamin D supplements as clinically needed. In addition they received vitamin B6 (pyridoxine hydrochloride) supplements (50 mg every other day). Control subjects were on a diet providing 30–32 kcal and 0.9–1.2 grams of protein/Kg/day, as assessed by dietary histories and urea excretion. Routine laboratory tests, acid–base, and electrolyte measurements were all normal.

The study was part of a larger protocol on amino acid and protein metabolism in CKD approved by the Ethical Committee of the Department of Internal Medicine of the University of Genoa (Ref. N.62006; Garibotto et al., 2006, 2015). All subjects were informed about the nature, purposes, procedures, and possible risks of the study before their informed consent was obtained. The procedures were in accordance with the Helsinki declaration.

### 2.1 Protocol

All studies were performed in the overnight, basal postabsorptive state. HD patients were studied after approximately 72 to 74 h from the last dialytic treatment. Briefly, a brachial artery and an ipsilateral retrograde cubital vein catheter were placed percutaneously. Triplicate sets of arterial and venous samples were taken at 20‐min intervals during a 60‐min period. Forearm blood flow was measured after each sample set.

### 2.2 Analytic procedures

Since phenylalanine is not metabolized in muscle, the measure of the net phenylalanine balance across the forearm represents the difference between protein synthesis and degradation. In the postabsorptive condition, as evaluated in the present study, the net balance of phenylalanine across the forearm (as an expression of net protein balance) is negative because protein degradation is greater than protein synthesis. During the study, blood samples were collected in heparinized syringes and immediately kept in ice until delivery to the laboratory. At the lab, blood samples were transferred into cooled ethylendiaminetetraacetate (EDTA) tubes, which were immediately centrifuged at 6000 rpm for 10 min at +4°C. Plasma was quickly separated from blood cells and stored at −80°C until assayed. Total Hcy concentrations were determined in triplicate in plasma samples by HPLC (Chadwick et al., 2000) within 1 year from sampling. Blood phenylalanine was determined by an amino acid analyzer. All other serum chemical measurements were determined by routine clinical chemistry laboratory procedures: folate and vitamin B12 were determined by competitive protein binding techniques. Total folate concentrations determined by protein binding are approximately 9% lower than results obtained with LC/MS/MS.

### 2.3 Calculations

All the results are corrected × 100 ml forearm volume. Forearm volume was measured in a large plastic cylinder by water displacement from the tip of the arterial catheter to the upper edge of the wrist cuff. Blood flow was expressed as ml/min.100 ml forearm volume (Garibotto et al., 2006, 2015). The exchange of Hcy and phenylalanine across the forearm was calculated by Fick's principle:((Xa)‐(Xv))x plasma flow, where Xa and Xv are the concentrations of Hcy in arterial and venous plasma, respectively. The results are expressed as nmol/min.100 ml forearm.

### 2.4 Statistical analyses

All data are presented as the mean ± SEM. Statistical analysis was performed using the two‐tailed t test to compare arterial with venous data. Linear regression and correlation were used to evaluate the relationships between muscle Hcy exchange and plasma folate, vitamin B12, IL‐6 and net muscle protein balance. Among cytokines, we focused on IL‐6 because it is secreted by skeletal muscle and may act locally (Garibotto et al., 2006). Non‐Gaussian distributed variables were Log transformed before analysis. Statistical analysis was performed with the GraphPad Prism Statistical Package (San Diego).

## 3 RESULTS

No significant differences among study groups were observed for mean plasma folate (13 ± 3, 11 ± 2 and 9 ± 2 ng/mL in CKD, HD and control subjects, respectively, *p* = NS). Also mean vitamin B12 levels (908 ± 37, 963 ± 58 and 800 ± 67 pg/mL in CKD, HD and control subjects, respectively, *p* = NS) were similar among groups.

**FIGURE 2:**
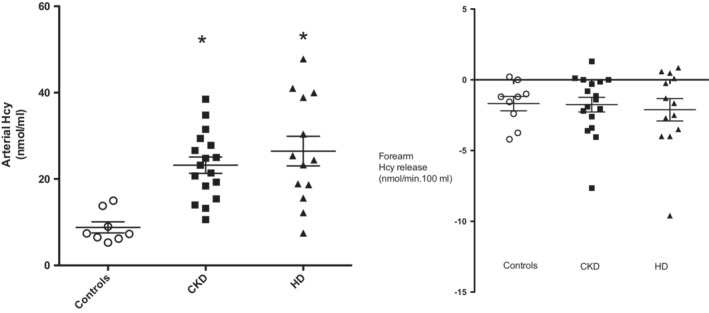
Homocysteine (Hcy) arterial levels and Hcy exchange across the forearm in CKD (*n* = 17), HD (*n* = 14) and control (*n* = 9) subjects. Statistically significant from controls:* *p* < 0.05–0.03.

Average Hcy levels, as well as their exchange rates across the forearm, are reported in Figure 2. Arterial Hcy levels were within the normal range (Bostom & Culleton, 1999) in controls (average 9 ± 2.5 nmoL/mL) and were ~ 2.5 folds increased in CKD and HD patients (23 ± 1.9 and 26 ± 3.3 nmoL/mL, respectively, *p* < 0.05–0.03). Both in controls and in patients Hcy levels in the deep forearm vein were consistently greater (+~7%, *p* < 0.05–0.01) than the corresponding arterial level, indicating the occurrence of Hcy release from muscle. The release of Hcy from the forearm was similar among groups (−1.8 ± 0.5, −2.1 ± 0.8 and −1.7 ± 0.5 nmol/min.100 ml forearm in CKD, HD patients and controls, respectively; Figure 2).

**FIGURE 3:**
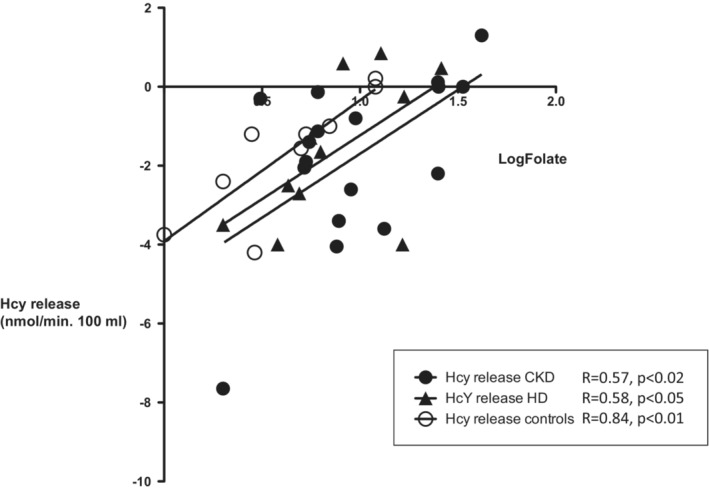
Relationships between the forearm exchange of homocysteine (Hcy) and plasma folate levels in CKD (*n* = 17), HD (*n* = 14), and control (*n* = 9) subjects.

To study the determinants of muscle Hcy metabolism we examined the relationships between muscle Hcy exchange and plasma folate, vitamin B12, IL‐6 and net muscle protein balance (Table 1). Muscle release of Hcy was inversely related to folate level in controls and both in CKD and HD patients (Table 1, Figure 3). The slopes were nonstatistically different (*p* = 0.98). Visually, the intercepts with the *x*‐axis (folate levels) were higher for CKD and HD patients vs. controls, suggesting resistance to folate in renal patients. However, the values were nonstatistically different (*p* = 0.19).

**TABLE 1:** Relationship between Hcy release from the forearm and potential determinants of Hcy metabolism in muscle in kidney patients (both CKD and HD patients)

	*r*	*p*
Logfolate (ng/ml)	−0.420	0.024
Vitamin B12 (pg/ml)	0.080	NS
Muscle Net protein balance (nmol/min.100 L)	0.180	NS
Interleukin‐6 (pg/ml)	0.060	NS

**FIGURE 4:**
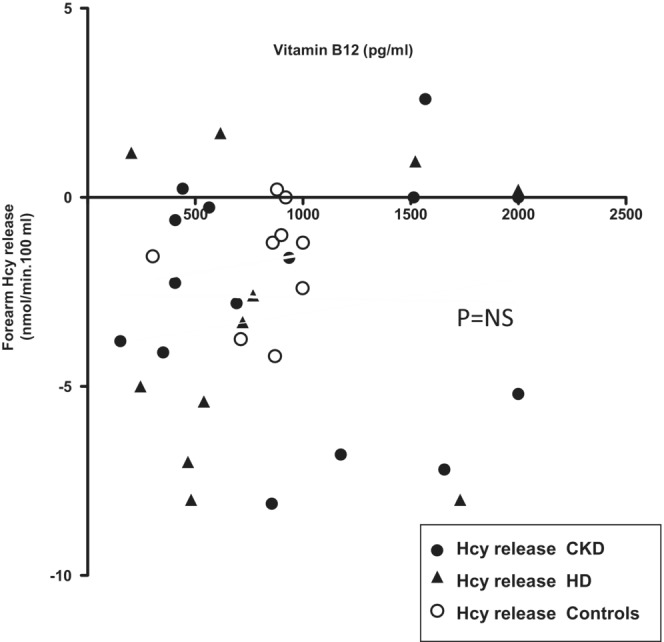
Relationship between the forearm exchange of homocysteine (Hcy) and plasma vitamin B12 levels in CKD (*n* = 15), HD (*n* = 10), and control (*n* = 9) subjects.

No associations between vitamin B12 levels and forearm Hcy release was observed for both patients and controls (Table 1, Figure 4). No relationship was also observed between Il‐6 levels and the forearm release of Hcy neither when CKD and HD patients were analyzed individually (*r* = 0.03 and *r* = 0.02 for CKD and HD patients, respectively) nor when data were pooled (*r* = 0.06, *p* = NS). Similarly, there was no relationship between forearm muscle protein net balance and Hcy release (*r* = 0.11 and *r* = 0.10, for CKD and HD patients, respectively, *p* = NS).

**FIGURE 5:**
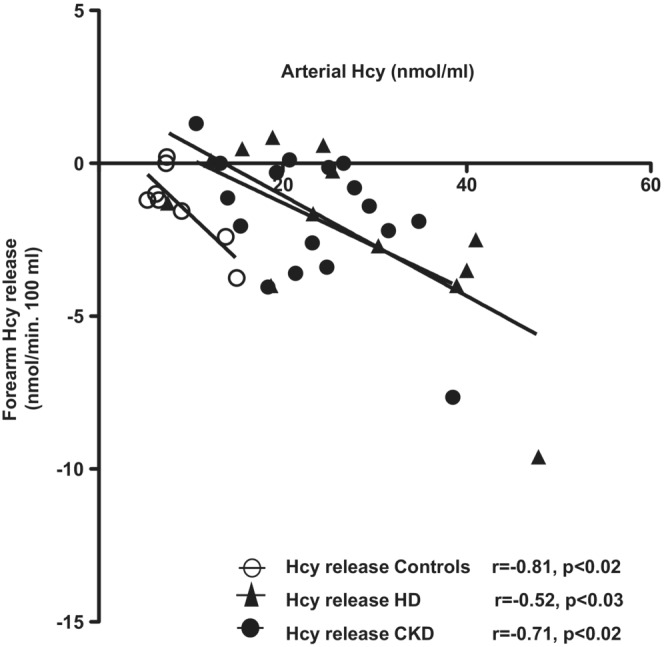
Relationships between arterial homocysteine (Hcy) levels and Hcy forearm exchange in CKD (*n* = 17), HD (*n* = 14), and control (*n* = 9) subjects.

In control subjects arterial Hcy was related to its release from muscle (*r* = −0.81, *p* < 0.02; Figure 5). Also, Arterial Hcy was also related to the release of the same amino acid both in CKD and HD patients (*r* = −0.52; *p* < 0.03 and *r* = −0.71; *p* < 0.02, respectively), suggesting that release from peripheral tissues plays a major role on plasma Hcy levels. The slopes were not significantly different from controls. The intercept of the *x*‐axis of pooled data was borderline (*p* < 0.075) statistically significant from controls.

## 4 DISCUSSION

There are several observations indicating that sulfur amino acid levels are altered in the uremic muscle, suggesting abnormal methionine metabolism. As shown by early studies on intracellular amino acid concentration in uremic patients, methionine is normal, while cysteine is increased and taurine is present in low concentration (Bergstrom et al., 1990; Lindholm et al., 1989). The current study used the forearm kinetics of Hcy to better understand the role of skeletal muscle in Hcy metabolism and to unravel some of the interactions between muscle Hcy exchange and its potential modulators, such as net protein balance (as a source of methionine), IL‐6 and folate/B12 circulating levels. Three major observations are made from this study. First, in accordance with our previous observation in healthy subjects (Garibotto et al., 2003), in patients with CKD the skeletal muscle releases Hcy in the circulation, a finding, which is in keeping with limited remethylation in human muscle. Second, as a new finding, our data suggest that both in patients with CKD, as well as in the normal condition, the Hcy export from peripheral tissues is an important contributor to Hcy circulating levels; of note, in CKD, the Hcy release from muscle is quantitatively similar to the normal condition. Third, the Hcy export output from muscle is highly variable according to folate plasma levels.

In patients with CKD, blood Hcy levels are inversely related to GFR over wide range of kidney function, which suggests that kidney uptake and/or metabolism plays a major role in Hcy removal (Bostom & Culleton, 1999). As a matter of fact, a decreased whole body transmethylation rate is observed in patients even in patients with moderate degree CKD (Tessari et al., 2005). In addition, decreased whole body remethylation rates have been also observed in CKD5, HD‐treated patients (van Guldener et al., 1999). Furthermore, the human kidney plays a major role on the removal of S‐adenosylhomocysteine (SAH), a potent inhibitor of methylation reactions, which likely accounts for increased SAH levels in CKD (Garibotto et al., 2009). However, Hcy increase in blood can also be promoted by factors that are often observed in CKD, such as deficiencies of folate and vitamin B_12_ and/or genetic variants of several enzymes of folate and one‐carbon pool metabolism (Lee et al., 1999; Perna & Ingrosso, 2019). Our study shows that the release of Hcy from skeletal muscle to the blood compartment is not increased in CKD; however, muscle is extremely sensitive to low folate levels, with large increases in Hcy export observed in patients with subnormal plasma folate. These findings are in accordance with the strong influence of folate on MS in muscle cells (Chen et al., 1997). Although nonstatistically significant, in CKD, lack of Hcy release from muscle is observed at higher‐than‐normal plasma folate levels, suggesting the occurrence of altered Hcy inflow/outflow from muscle or CKD‐related resistance of the folate‐dependent muscle Hcy remethylation pathway. However, since Hcy muscle inflow is high (Fiona et al., 2009), a defective Hcy transport in muscle in uremia cannot be ruled out from our study. In most normal subjects, a folate dose of 400 to 600 μg produces a prompt fall of 20% to 30% in total plasma Hcy concentrations; in contrast, patients with ESRD are more resistant to this Hcy‐lowering action of folic acid and even the use of pharmacological doses of folic acid or reduced folates does not normalize completely circulating Hcy levels (Ducloux et al., 2002; Massy, 2003; Sunder‐Plassmann et al., 2000).

More commonly occurring vitamin deficiencies in maintenance dialysis patients include those for vitamin C, folate and pyridoxine (Kalantar‐Zadeh & Kopple, 2003). Patients studied here received pyridoxine supplementation; therefore, the role of pyridoxine on Hcy muscle metabolism could not be addressed. In addition, the normal vitamin B12 levels observed in patients studied here (no subject had a B12 serum level <150 pg/mL; Hunt et al., 2014) may have accounted for a lack of association between vitamin B12 levels and Hcy muscle release from muscle.

Methionine provides a source of methyl groups that can reduce the need for C‐1 transfers via folate to methylate homocysteine (Ducker & Rabinowitz, 2017). In the postabsorptive state skeletal muscle protein degradation is a major source of intracellular methionine. In patients studied here, net protein balance was to a similar extent negative in patients and control subjects, suggesting that in CKD the intracellular methionine supply was similar to controls. In addition, no association was observed between net protein balance and forearm Hcy release.

Hcy release from tissues can be promoted by oxidative stress and inflammation (Giustarini et al., 2009). However, in our study, no association between Hcy release from muscle and IL‐6 levels was observed. This suggests that methionine metabolism in muscle is less sensitive to inflammation than in other tissues. However, a role of the inflammasome on Hcy muscle metabolism cannot be ruled out, since the inflammasome components were not evaluated in our study.

Clearance studies in healthy adult humans estimate that 1.2 mmol of Hcy, or approximately 5% to 10% of the total daily cellular production, is delivered daily to the plasma compartment; this figure increases by a factor of 20 × in vitamin‐depleted subjects (Refsum et al., 1998). Considering that the mean fraction of forearm made up of muscle tissue is approximately 0.6 (Yki‐Jarvinen et al., 1987), that forearm blood flow in forearm muscle is approximately 60% to 70% of total flow, that muscle is on the average 40% of body weight, the estimate of the amount of Hcy released into the circulation gives a figure of about 790 umol/day, that is, 66% of the calculated Hcy daily delivery of the plasma compartment (Refsum et al., 1998). This makes the skeletal muscle the major site for export of Hcy to extracellular fluids.

Whole body transmethylation, remethylation and transsulfuration rates have been already studied in healthy subjects (Fukagawa et al., 1998, 2000; MacCoss et al., 2001) and in renal patients (Guttmorsen et al., 1997; Stam et al., 2003; van Guldener et al., 1999), but no data regarding methionine kinetics in muscle are available. According to our data Hcy release from muscle account as for 5%–10% of whole body remethylation (Davis et al., 2004), suggesting that the amount of Hcy escaping from the intracellular remethylation pathway is small. However, studies using systemic isotope infusion associated to limb amino acid kinetics might better quantify this estimate.

Our data also suggest that loss of muscle mass may cause a decrease in Hcy levels. This is in accordance with data showing that plasma Hcy is positively associated with markers of protein‐energy nutritional status, including somatic indexes, in maintenance dialysis patients (Kalantar‐Zadeh et al., 2003; Suliman et al., 2001). In addition data from our study, that show evidence of a role of muscle in Hcy metabolism, may offer a new paradigm for the study of sarcopenia and CKD‐related cachexia. Elevated levels of Hcy are associated with frailty, skeletal muscle malfunctioning, metabolic injury, and mortality (Kado et al., 2002; Veeranki & Tyagi, 2013). High Hcy might cause wasting by lowering ischemic skeletal muscle responses and angiogenesis through decline in PGC‐1α function (Veeranki et al., 2014). More recently, it has been shown that high Hcy upregulates mitophagy in skeletal muscle remodeling via epigenetic regulation (Singh et al., 2022) an effect that can be corrected by hydrogen sulfide.

### 4.1 Study limitations

In this study, the control subjects did not receive vitamin B6 or calcitriol supplements; therefore, the vitamin supplementation in patients might have influenced both Hcy levels and metabolism. In addition, folate assessment was limited to one single serum folate determination, which cannot distinguish between a transitory decrease in dietary folate intake and a chronic deficiency state. Similarly the use of a single serum cobalamin may be neither sensitive nor specific for cobalamin deficiency. Finally, this study may result not enough sensitive to detect small changes in forearm Hcy exchange occurring in CKD, since Hcy presents a 30% lower enrichment in the forearm vein with respect to phenylalanine. However, increasing sample size was not feasible in this study, which was based on retrospective data collection.

## 5 CONCLUSION

In conclusion, our study shows that skeletal muscle is a major provider of Hcy to the systemic circulation both in the normal condition and in patients with CKD. Our study also suggests that extensive Hcy muscle shuttling to the organs, which possess the entire methionine cycle enzymes is a necessary homeostatic response to maintain Hcy levels. Folate plasma levels appear to be a major determinant of muscle Hcy release, suggesting that lack of transsulfuration and hindered remethylation pathway are critical for Hcy escape from muscle. Data provided by this work could be useful to the understanding of the sites regulating 1‐C pool metabolism and the mechanisms leading to muscle protein wasting, observed in many systemic as well as organ diseases.

## AUTHOR CONTRIBUTIONS

Giacomo Garibotto and Antonella Sofia designed the study and performed the experiments, Alessando Valli performed the Hcy analysis, Daniela Picciotto and Daniela Verzola analyzed the data and made the figures, Francesca Costigliolo, Michela Saio and Pasquale Esposito drafted the manuscript, Alessando Valli and Francesca Viazzi edited and revised the manuscripts. All authors have read and approved the final version of the manuscript.

## FUNDING INFORMATION

This study was supported by grants from the Ministero dell’ Università e della Ricerca Scientifica e Tecnologica PRIN, Iyali.

## CONFLICT OF INTEREST

No conflicts of interest, financial or otherwise, are declared by the authors.

## ETHICS STATEMENT

All experimental procedures were reviewed and approved by the Ethical Committee of the Department of Internal Medicine of the University of Genoa. All subjects were informed about the nature, purposes, procedures, and possible risks of the study before their informed consent was obtained. The procedures were in accordance with the Helsinki declaration.
